# Service user experience of remote consultations during COVID-19 in an older adult community mental health setting

**DOI:** 10.1192/bjo.2021.109

**Published:** 2021-06-18

**Authors:** Darena Dineva, Sukran Altun, Tahiya Twaha, Juliette Brown

**Affiliations:** East London NHS Foundation Trust

## Abstract

**Aims:**

The COVID-19 pandemic has had a significant effect on our ability to communicate face-to-face with patients freely. Similar to other medical specialities and general practice (1), to continue providing a service for our service users, we employed other means of communication including telephone and video consultations (although face-to-face consultations were available for high clinical concern and/or identified risk). We set out to explore the acceptability of remote consultation for service users of an older adult (>65 years) Community Mental Health Team (CMHT).

Reference: *BMJ* 2020;371:m3945

**Method:**

A total of 34 service users were selected randomly from the CMHT caseload (9% of total 372 caseload). 4 clinicians were involved in collecting data between July and October 2020. We used our trust's (East London Foundation Trust) standard questionnaire on patient satisfaction and contacted individuals by telephone to complete the survey.

**Result:**

For both questions of ‘I felt listened to by the team’ and ‘I feel I have been given enough information regarding my care’ 17 (50%) responders ‘agreed’ with this statement and an additional 13 (38%) ‘strongly agreed’ (total of 88%). For the statement ‘I feel involved in decisions about my care’ 16 (47%) responders ‘agreed’ and a further 11 (32%) responders ‘strongly agreed’ with this statement. The statement ‘The professionals involved in my care talk to each other and we all work as a team’ had 15 (44%) responders ‘agree’ and 13 (38%) responders ‘strongly agree’. When asked ‘If you experienced telephone/video sessions, were these helpful?’ 31 responders said ‘yes’.

**Conclusion:**

Overall most responders agreed or strongly agreed that they felt listened to, were given enough information about their care, felt involved in decisions about their care and that they worked as a team with the professionals involved in their care. 91% of responders felt that the video/telephone sessions had been helpful. These data have provided reassurance that telemedicine methods were a useful substitute for face-to-face consultations during the early stages of the pandemic. However this was a small scale study. This study cannot tell us about the experience after the initial 6 months of the pandemic, how often it would be optimal for people have face to face reviews, or whether satisfaction with telemedicine altered over a longer period. We were also not able to tell whether the experience varied for those who had less robust and longstanding relationships with their clinicians.

